# Pairwise Maximum Entropy Models for Studying Large Biological Systems: When They Can Work and When They Can't

**DOI:** 10.1371/journal.pcbi.1000380

**Published:** 2009-05-08

**Authors:** Yasser Roudi, Sheila Nirenberg, Peter E. Latham

**Affiliations:** 1 Gatsby Computational Neuroscience Unit, University College London, London, United Kingdom; 2 Department of Physiology and Biophysics, Weill Medical College of Cornell University, New York, United States of America; Indiana University, United States of America

## Abstract

One of the most critical problems we face in the study of biological systems is building accurate statistical descriptions of them. This problem has been particularly challenging because biological systems typically contain large numbers of interacting elements, which precludes the use of standard brute force approaches. Recently, though, several groups have reported that there may be an alternate strategy. The reports show that reliable statistical models can be built without knowledge of all the interactions in a system; instead, pairwise interactions can suffice. These findings, however, are based on the analysis of small subsystems. Here, we ask whether the observations will generalize to systems of realistic size, that is, whether pairwise models will provide reliable descriptions of true biological systems. Our results show that, in most cases, they will not. The reason is that there is a crossover in the predictive power of pairwise models: If the size of the subsystem is below the crossover point, then the results have no predictive power for large systems. If the size is above the crossover point, then the results may have predictive power. This work thus provides a general framework for determining the extent to which pairwise models can be used to predict the behavior of large biological systems. Applied to neural data, the size of most systems studied so far is below the crossover point.

## Author Summary

Biological systems are exceedingly complicated: They consist of a large number of elements, those elements interact in nonlinear and highly unpredictable ways, and collective interactions typically play a critical role. It would seem surprising, then, that one could build a quantitative description of biological systems based only on knowledge of how pairs of elements interact. Yet, that is what a number of studies have found. Those studies, however, focused on relatively small systems. Here, we ask the question: Do their conclusions extend to large systems? We show that the answer depends on the size of the system relative to a crossover point: Below the crossover point the results on the small system have no predictive power for large systems; above the crossover point the results on the small system may have predictive power. Moreover, the crossover point can be computed analytically. This work thus provides a general framework for determining the extent to which pairwise models can be used to predict the behavior of large biological systems. It also provides a useful heuristic for designing experiments: If one is interested in understanding truly large systems via pairwise interactions, then make sure that the system one studies is above the crossover point.

## Introduction

Many fundamental questions in biology are naturally treated in a probabilistic setting. For instance, deciphering the neural code requires knowledge of the probability of observing patterns of activity in response to stimuli [1]; determining which features of a protein are important for correct folding requires knowledge of the probability that a particular sequence of amino acids folds naturally [2],[3]; and determining the patterns of foraging of animals and their social and individual behavior requires knowledge of the distribution of food and species over both space and time [4]–[6].

Constructing these probability distributions is, however, hard. There are several reasons for this: i) biological systems are composed of large numbers of elements, and so can exhibit a huge number of configurations—in fact, an exponentially large number, ii) the elements typically interact with each other, making it impossible to view the system as a collection of independent entities, and iii) because of technological considerations, the descriptions of biological systems have to be built from very little data. For example, with current technology in neuroscience, we can record simultaneously from only about 100 neurons out of approximately 100 billion in the human brain. So, not only are we faced with the problem of estimating probability distributions in high dimensional spaces, we must do this based on a small fraction of the neurons in the network.

Despite these apparent difficulties, recent work has suggested that the situation may be less bleak than it seems, and that an accurate statistical description of systems can be achieved without having to examine all possible configurations [2], [3], [7]–[11]. One merely has to measure the probability distribution over pairs of elements and use those to build the full distribution. These “pairwise models” potentially offer a fundamental simplification, as the number of pairs is quadratic in the number of elements, not exponential. However, support for the efficacy of pairwise models has, necessarily, come from relatively small subsystems—small enough that the true probability distribution could be measured experimentally [7]–[9],[11]. While these studies have provided a key first step, a critical question remains: will the results from the analysis of these small subsystems extrapolate to large ones? That is, if a pairwise model predicts the probability distribution for a subset of the elements in a system, will it also predict the probability distribution for the whole system? Here we find that, for a biologically relevant class of systems, this question can be answered quantitatively and, importantly, generically—independent of many of the details of the biological system under consideration. And the answer is, generally, “no.” In this paper, we explain, both analytically and with simulations, why this is the case.

## Results

### The extrapolation problem

**Figure 1:**
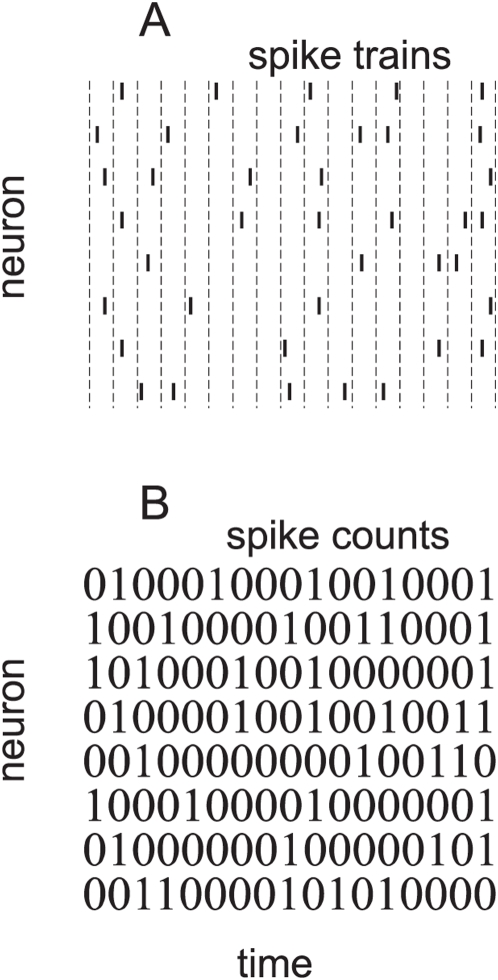
Transforming spike trains to spike count. (A) Spike rasters. Tick marks indicate spike times; different rows correspond to different neurons. The horizontal dashed lines are the bin boundaries. (B) Spike count in each bin. In this example the bins are small enough that there is at most one spike per bin, but this is not necessary—one could use bigger bins and have larger spike counts.

To gain intuition into the extrapolation problem, let us consider a specific example: neuronal spike trains. Fig. 1A shows a typical spike train for a small population of neurons. Although the raw spike times provide a complete description, they are not a useful representation, as they are too high-dimensional. Therefore, we divide time into bins and re-represent the spike train as 0 s and 1 s: 0 if there is no spike in a bin; 1 otherwise (Fig. 1B) [7]–[9],[11]. For now we assume that the bins are independent (an assumption whose validity we discuss below, and in more detail in the section “Is there anything wrong with using small time bins?”). The problem, then, is to find 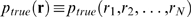 where 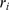 is a binary variable indicating no spike (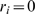) or one or more spikes (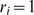) on neuron 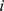. Since this, too, is a high dimensional problem (though less so than the original spike time representation), suppose that we instead construct a pairwise approximation to 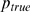, which we denote 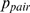, for a population of size 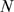. (The pairwise model derives its name from the fact that it has the same mean and pairwise correlations as the true model; see Eq. (15).) Our question, then, is: if 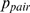 is close to 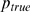 for small 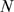, what can we say about how close the two distributions are for large 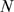
?

**Figure 2:**
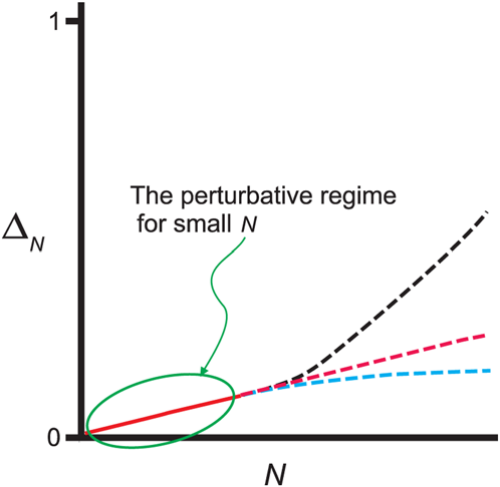
Cartoon illustrating the dependence of 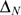 on 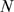. For small 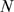 there is always a perturbative regime in which 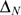 increases linearly with 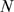 (solid red line). When 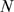 becomes large, 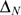 may continue increasing with 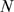 (red and black dashed lines) or it may plateau (cyan dashed line), depending on 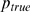. The observation that 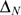 increases linearly with 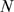 does not, therefore, provide much, if any information about the large 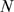 behavior.

To answer this question quantitatively, we need a measure of distance. The measure we use, denoted 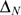, is defined in Eq. (3) below, but all we need to know about it for now is that if 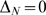 then 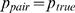, and if 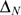 is near one then 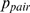 is far from 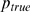. In terms of 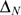, our main results are as follows. First, for small 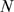, in what we call the perturbative regime, 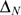 is proportional to 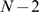. In other words, as the population size increases, the pairwise model becomes a worse and worse approximation to the true distribution. Second, this behavior is entirely generic: for small 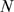, 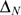 increases linearly, no matter what the true distribution is. This is illustrated schematically in Fig. 2, which shows the generic behavior of 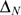. The solid red part of the curve is the perturbative regime, where 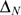 is a linearly increasing function of 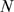; the dashed curves show possible behavior beyond the perturbative regime.

These results have an important corollary: if one does an experiment and finds that 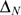 is increasing linearly with 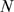, then one has no information at all about the true distribution. The flip side of this is more encouraging: if one can measure the true distribution for sufficiently large 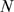 that 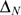 saturates, as for the dashed blue line in Fig. 2, then there is a chance that extrapolation to large 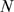 is meaningful. The implications for the interpretation of experiments is, therefore, that one can gain information about large 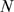 behavior only if one can analyze data past the perturbative regime.

Under what conditions is a subsystem in the perturbative regime? The answer turns out to be simple: the size of the system, 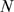, times the average probability of observing a spike in a bin, must be small compared to 1. For example, if the average probability is 1/100, then a system will be in the perturbative regime if the number of neurons is small compared to 100. This last observation would seem to be good news: if we divide the spike trains into sufficiently small time bins and ignore temporal correlations, then we can model the data very well with a pairwise distribution. The problem with this, though, is that temporal correlations can be ignored only when time bins are large compared to the autocorrelation time. This leads to a kind of catch-22: pairwise models are guaranteed to work well (in the sense that they describe spike trains in which temporal correlations are ignored) if one uses small time bins, but small time bins is the one regime where ignoring temporal correlations is not a valid approximation.

In the next several sections we quantify the qualitative picture presented above: we write down an explicit expression for 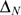, explain why it increases linearly with 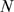 when 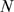 is small, and provide additional tests, besides assessing the linearity of 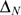, to determine whether or not one is in the perturbative regime.

### Quantifying how well the pairwise model explains the data

A natural measure of the distance between 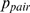 and 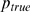 is the Kullback-Leibler (KL) divergence [12], denoted 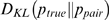 and defined as
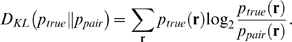
(1)


The KL divergence is zero if the two distributions are equal; otherwise it is nonzero.

Although the KL divergence is a very natural measure, it is not easy to interpret (except, of course, when it is exactly zero). That is because a nonzero KL divergence tells us that 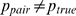, but it does not give us any real handle on how good, or bad, the pairwise model really is. To make sense of the KL divergence, we need something to compare it to. A reasonable reference quantity, used by a number of authors [7]–[9], is the KL divergence between the true distribution and the independent one, the latter denoted 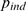. The independent distribution, as its name suggests, is a distribution in which the variables are taken to be independent,
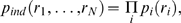
(2)where 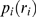 is the distribution of the response of the 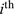 neuron, 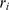. With this choice for a comparison, we define a normalized distance measure—a measure of how well the pairwise model explains the data—as
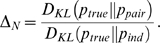
(3)


Note that the denominator in this expression, 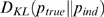, is usually referred to as the multi-information [7],[13],[14].

The quantity 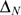 lies between 0 and 1, and measures how well a pairwise model does relative to an independent model. If it is 0, the pairwise model is equal to the true model (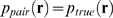); if it is near 1, the pairwise model offers little improvement over the independent model; and if it is exactly 1, the pairwise model is equal to the independent model (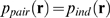), and so offers no improvement.

How do we attach intuitive meaning to the two divergences 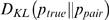 and 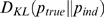
? For the latter, we use the fact that, as is easy to show,
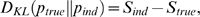
(4)where 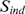 and 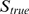 are the entropies [15],[16] of 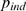 and 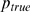, respectively, defined, as usual, to be 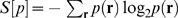. For the former, we use the definition of the KL divergence to write
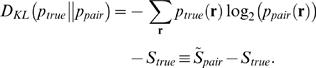
(5)


**Figure 3:**
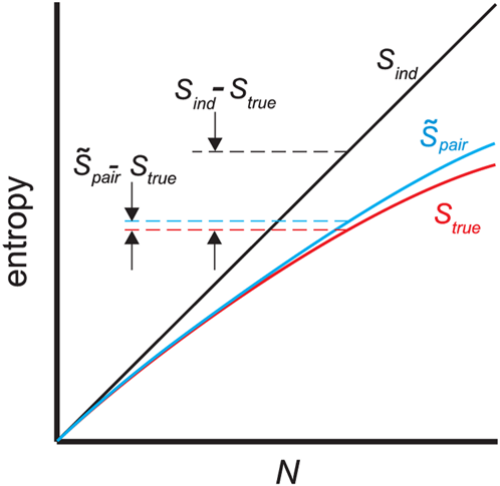
Schematic plot of 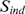 (black line), 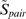 (cyan line) and 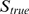 (red line). The better the pairwise model, the closer 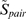 is to 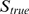. This is reflected in the normalized distance measure, 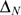, which is the distance between the red and cyan lines divided by the distance between the red and black lines.

The quantity 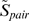 has the flavor of an entropy, although it is a true entropy only when 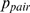 is maximum entropy as well as pairwise (see Eq. (6) below). For other pairwise distributions, all we need to know is that 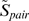 lies between 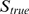 and 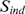. A plot illustrating the relationship between 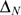, the two entropies 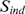 and 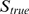, and the entropy-like quantity 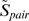, is shown in Fig. 3.

Note that for pairwise maximum entropy models (or maximum entropy models for short), 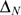 has a particularly simple interpretation, since in this case 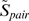 really is an entropy. Using 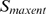 to denote the pairwise entropy of a maximum entropy model, for this case we have
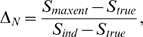
(6)as is easy to see by inserting Eqs. (4) and (5) into (3). This expression has been used previously by a number of authors [7],[9].

### 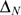 in the perturbative regime

The extrapolation problem discussed above is the problem of determining 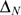 in the large 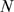 limit. This is hard to do in general, but there is a perturbative regime in which it is possible. The small parameter that defines this regime is the average number of spikes produced by the whole population of neurons in each time bin. It is given quantitatively by 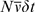 where 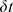 is the bin size and 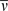 the average firing rate,
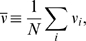
(7)with 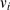 the firing rate of neuron 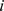.

The first step in the perturbation expansion is to compute the two quantities that make up 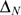: 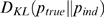 and 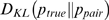. As we show in the section “Perturbative Expansion” (Methods), these are given by
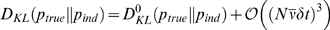
(8a)

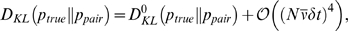
(8b)where
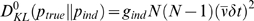
(9a)

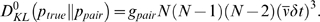
(9b)


Here and in what follows we use 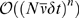 to denote terms that are proportional to 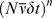 in the limit 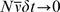. The 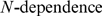 in Eq. (9a) has been noted previously [7], although the authors did not compute the prefactor, 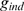.

The prefactors 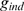 and 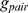, which are given explicitly in Eqs. (42) and (44), depend on the low order statistics of the spike trains: 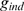 depends on the second order normalized correlation coefficients, 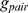 depends on the second and third order normalized correlation coefficients (the normalized correlation coefficients are defined in Eq. (16) below), and both depend on the firing rates of the individual cells. The details of that dependence, however, are not important for now; what is important is that 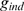 and 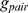 are independent of 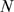 and 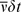 (at least on average; see next section).

Inserting Eq. (8) into Eq. (3) (into the definition of 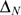) and using Eq. (9), we arrive at our main result,
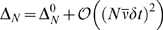
(10a)

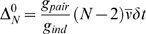
(10b)


Note that in the regime 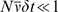, 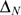 is necessarily small. This explains why, in an analytic study of non-pairwise model in which 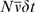 was small, Shlens et al. found that 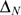 was rarely greater than 0.1 [8].

We refer to quantities with a superscript zero as “zeroth order.” Note that, via Eqs. (4) and (5), we can also define zeroth order entropies,
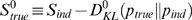
(11a)

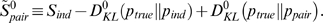
(11b)


**Figure 4:**
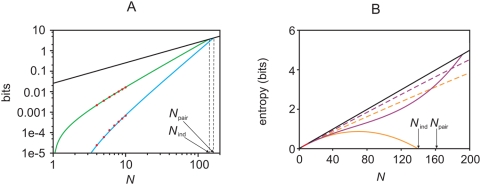
Cartoon showing extrapolations of the zeroth order KL divergences and entropies (see Eqs. (9) and (11)). These extrapolations illustrate why the two natural quantities derived from them, 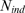 and 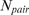, occur beyond the point at which the extrapolation is meaningful. (A) Extrapolations on a log-log scale. Black: 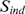; green: 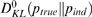; cyan: 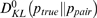. The red points are the data. The points 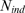 and 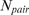 label the intersections of the two extrapolations with the independent entropy, 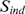. (B) Extrapolation of the entropies rather than the KL divergences, plotted on a linear-linear scale. The data, again shown in red, is barely visible in the lower left hand corner. Black: 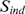; solid orange: 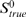; solid maroon: 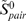. The dashed orange and maroon lines are the extrapolations of the true entropy and true pairwise “entropy”, respectively.

These quantities are important primarily because differences between them and the actual entropies indicate a breakdown of the perturbation expansion (see in particular Fig. 4 below).

Assuming, as discussed in the next section, that 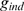 and 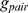 are approximately independent of 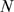, 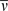, and 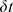, Eq. (10) tells us that 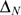 scales linearly with 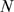 in the perturbative regime—the regime in which 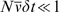. The key observation about this scaling is that it is independent of the details of the true distribution, 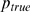. This has a very important consequence, one that has major implications for experimental data: if one does an experiment with small 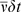 and finds that 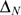 is proportional to 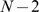, then the system is, with very high probability, in the perturbative regime, and one does not know whether 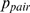 will remain close to 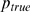 as 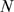 increases. What this means in practical terms is that if one wants to know whether a particular pairwise model is a good one for large systems, it is necessary to consider values of 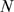 that are significantly greater than 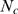, where
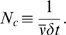
(12)


We interpret 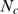 as the value at which there is a crossover in the behavior of the pairwise model. Specifically, if 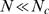, the system is in the perturbative regime and the pairwise model is not informative about the large 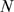 behavior, whereas if 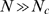, the system is in a regime in which it may be possible to make inferences about the behavior of the full system.

### The prefactors, 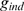 and 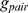


**Figure 5:**
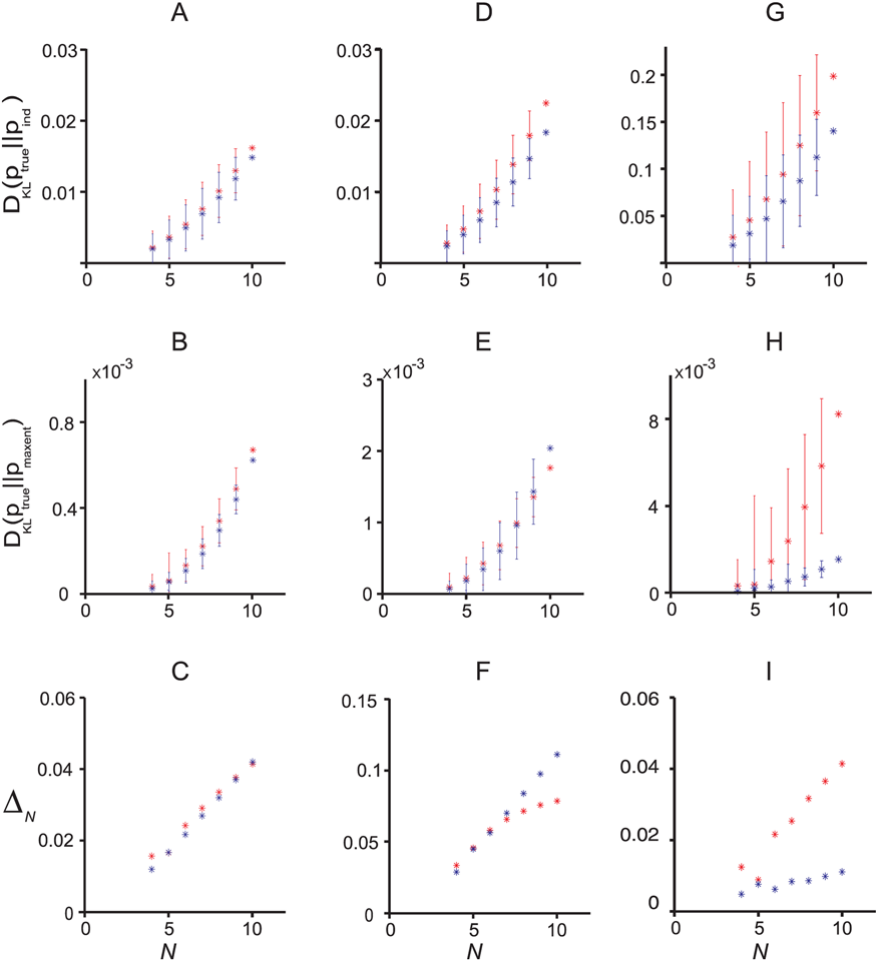
The 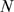 dependence of the KL divergences and the normalized distance measure, 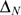. Data was generated from a third order model, as explained in the section “Generating synthetic data” (Methods), and fit to pairwise maximum entropy models and independent models. All data points correspond to averages over marginalizations of the true distribution (see text for details). The red points were computed directly using Eqs. (1), (3) and (4); the blue points are the zeroth order estimates, 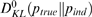, 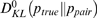, and 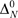, in rows 1, 2 and 3, respectively. The three columns correspond to 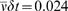, 0.029, and 0.037, from left to right. (A, B, C) (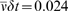). Predictions from the perturbative expansion are in good agreement with the measurements up to 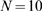, indicating that the data is in the perturbative regime. (D, E, F) (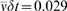). Predictions from the perturbative expansion are in good agreement with the measurements up to 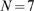, indicating that the data is only partially in the perturbative regime. (G, H, I) (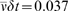). Predictions from the perturbative expansion are not in good agreement with the measurements, even for small 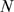, indicating that the data is outside the perturbative regime.

As we show in Methods (see in particular Eqs. (42) and (44)), the prefactors 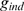 and 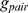 depend on which neurons out of the full population are used. Consequently, these quantities fluctuate around their true values (in the sense that different subpopulations produce different values of 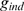 and 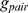), where “true” refers to an average over all possible 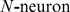 sub-populations. Here we assume that the 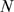 neurons are chosen randomly from the full population, so any set of 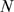 neurons provides unbiased estimates of 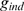 and 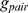. In our simulations, the fluctuations were small, as indicated by the small error bars on the blue points in Fig. 5. However, in general the size of the fluctuations is determined by the range of firing rates and correlation coefficients, with larger ranges producing larger fluctuations.

Because 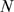 does not affect the mean values of 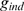 and 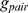, it is reasonable to think of these quantities—or at least their true values—as being independent of 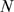. They are also independent of 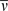, again modulo fluctuations. Finally, as we show in the section “Bin size and the correlation coefficients” (Methods), 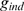 and 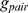 are independent of 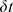 in the limit that 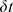 is small compared to the width of the temporal correlations among neurons. We will assume this limit applies here. In sum, then, to first approximation, 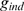 and 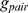 are independent of our three important quantities: 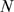, 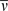, and 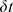. Thus, we treat them as effectively constant throughout our analysis.

### The dangers of extrapolation

Although the behavior of 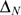 in the perturbative regime does not tell us much about its behavior at large 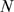, it is possible that other quantities that can be calculated in the perturbative regime, 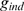, 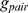, and 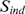 (the last one exactly), are informative, as others have suggested [7]. Here we show that this is not the case—they also are uninformative.

The easiest way to relate the perturbative regime to the large 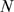 regime is to ignore the corrections in Eqs. (8a) and (8b), extrapolate the expressions for the zeroth order terms, and ask what their large 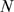 behavior tells us. Generic versions of these extrapolations, plotted on a log-log scale, are shown in Fig. 4A, along with a plot of the independent entropy, 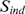 (which is necessarily linear in 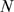). The first thing we notice about the extrapolations is that they do not, technically, have a large 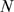 behavior: one terminates at the point labeled 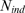, which is where 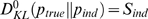 (and thus, via Eq. (0a), 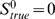; continuing the extrapolation implies negative true zeroth order entropy); the other at the point labeled 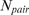, which is where 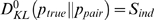 (and thus, via Eq. (5) and the fact that 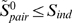, 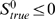).

Despite the fact that the extrapolations end abruptly, they still might provide information about the large 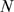 regime. For example, 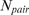 and/or 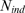 might be values of 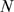 at which something interesting happens. To see if this is the case, in Fig. 4B we plot the naive extrapolations of 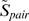 and 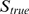 (that is, the zeroth order quantities given in Eq. (11), 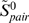 and 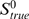), on a linear-linear plot, along with 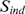. This plot contains no new information compared to Fig. 4A, but it does elucidate the meaning of the extrapolations. Perhaps its most striking feature is that the naive extrapolation of 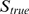 has a decreasing portion. As is easy to show mathematically, entropy cannot decrease with 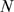 (intuitively, that is because observing one additional neuron cannot decrease the entropy of previously observed neurons). Thus, 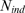, which occurs well beyond the point where the naive extrapolation of 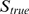 is decreasing, has essentially no meaning, something that has been pointed out previously by Bethge and Berens [10]. The other potentially important value of 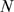 is 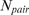. This, though, suffers from a similar problem: when 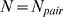, 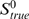 is negative.

How do the naively extrapolated entropies—the solid lines in Fig. 4B—compare to the actual entropies? To answer this, in Fig. 4B we show the true behavior of 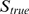 and 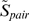 versus 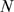 (dashed lines). Note that 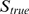 is asymptotically linear in 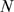, even though the neurons are correlated, a fact that forces 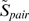 to be linear in 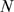, as it is sandwiched between 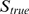 and 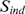. (The asymptotically linear behavior of 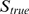 is typical, even in highly correlated systems. Although this is not always appreciated, it is easy to show; see the section “The behavior of the true entropy in the large *N* limit,” Methods.) Comparing the dashed and solid lines, we see that the naively extrapolated and true entropies, and thus the naively extrapolated and true values of 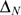, have extremely different behavior. This further suggests that there is very little connection between the perturbative and large 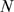 regimes.

In fact, these observations follow directly from the fact that 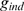 and 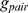 depend only on correlation coefficients up to third order (see Eqs. (42) and (44)) whereas the large 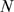 behavior depends on correlations at all orders. Thus, since 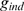 and 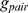 tell us very little, if anything, about higher order correlations, it is not surprising that they tell us very little about the behavior of 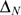 in the large 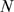 limit.

### Numerical simulations

To check that our perturbation expansions, Eqs. (8–10), are correct, and to investigate the regime in which they are valid, we performed numerical simulations. We generated, from synthetic data, a set of true distributions, computed the true distance measures, 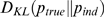, 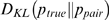, and 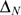 numerically, and compared them to the zeroth order ones, 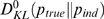, 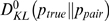, and 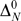. If the perturbation expansion is valid, then the true values should be close to the zeroth order values whenever 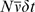 is small. The results are shown in Fig. 5, and that is, indeed, what we observed. Before discussing that figure, though, we explain our procedure for constructing true distributions.

The set of true distributions we used were generated from a third order model (so named because it includes up to third order interactions). This model has the form
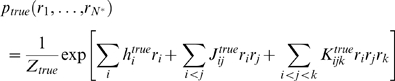
(13)where 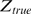 is a normalization constant, chosen to ensure that the probability distribution sums to 1, and the sums over 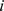, 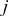 and 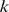 run from 1 to 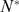. The parameters 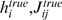 and 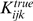 were chosen by sampling from distributions (see the section “Generating synthetic data,” Methods), which allowed us to generate many different true distributions. In all of our simulations we calculate the relevant quantities directly from Eq. (13) . Consequently, we do not have to worry about issues of finite data, as would be the case in realistic experiments.

For a particular simulation (corresponding to a column in Fig. 5), we generated a true distribution with 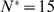, randomly chose 5 neurons, and marginalized over them. This gave us a 10-neuron true distribution. True distributions with 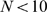 were constructed by marginalizing over additional neurons within our 10-neuron population. To achieve a representative sample, we considered all possible marginalizations (of which there are 10 choose 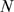, or 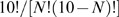). The results in Fig. 5 are averages over these marginalizations.

For neural data, the most commonly used pairwise model is the maximum entropy model. Therefore, we use that one here. To emphasize the maximum entropy nature of this model, we replace the label “*pair*” that we have been using so far with “*maxent*.” The maximum entropy distribution has the form
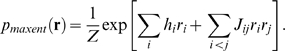
(14)


Fitting this distribution requires that we choose the 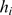 and 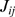 so that the first and second moments match those of the true distribution. Quantitatively, these conditions are
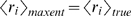
(15a)

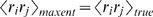
(15b)where the angle brackets, 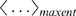 and 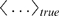, represent averages with respect to 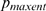 and 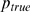, respectively. Once we have 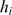 and 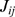 that satisfy Eq. (15), we calculate the KL divergences, Eqs. (1) and (4), and use those to compute 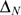.

The results are shown in Fig. 5. The rows correspond to our three quantities of interest: 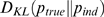, 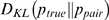, and 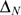 (top to bottom). The columns correspond to different values of 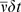, with the smallest 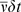 on the left and the largest on the right. Red circles are the true values of these quantities; blue ones are the zeroth order predictions from Eqs. (9) and (10b).

As suggested by our perturbation analysis, the smaller the value of 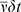, the larger the value of 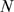 for which agreement between the true and zeroth order values is good. Our simulations corroborate this: the left column of Fig. 5 has 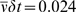, and agreement is almost perfect out to 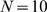; the middle column has 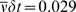, and agreement is almost perfect out to 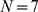; and the right column has 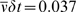, and agreement is not good for any value of 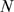. Note that the perturbation expansion breaks down for values of 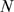 well below 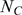 (defined in Eq.(12)): in the middle column of Fig. 5 it breaks down when 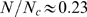, and in the right column it breaks down when 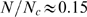. This is not, however, especially surprising, as the perturbation expansion is guaranteed to be valid only if 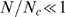.

These results validate the perturbation expansions in Eqs. (8) and (10), and show that those expansions provide sensible predictions—at least for some parameters. They also suggest a natural way to assess the significance of one's data: plot 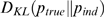, 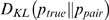, and 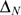 versus 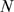, and look for agreement with the predictions of the perturbation expansion. If agreement is good, as in the left column of Fig. 5, then one is in the perturbative regime, and one knows very little about the true distribution. If, on the other hand, agreement is bad, as in the right column, then one is out of the perturbative regime, and it may be possible to extract meaningful information about the relationship between the true and pairwise models.

That said, the qualifier “at least for some parameters” is an important one. This is because the perturbation expansion is essentially an expansion that depends on the normalized correlation coefficients, and there is an underlying assumption that they don't exhibit pathological behavior. The 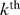 order normalized correlation coefficient for the distribution 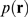, denoted 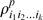, is written
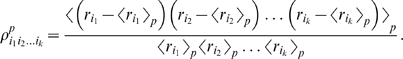
(16)A potentially problematic feature of the correlation coefficients is that the denominator is a product over mean activities. If the mean activities are small, the denominator can become very small, leading to very large correlation coefficients. Although our perturbation expansion is always valid for sufficiently small time bins (because the correlation coefficients eventually becomes independent of bin size; see the section “Bin size and the correlation coeffcients,” Methods), “sufficiently small” can depend in detail on the parameters. For instance, at the maximum population size tested (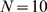) and for the true distributions that had 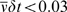, the absolute error of the prediction had a median of approximately 16%. However, about 11% of the runs had errors larger than 60%. Thus, the exact size of the small parameter at which the perturbative expansion breaks down can depend on the details of the true distribution.

### External fields and pairwise couplings have a simple dependence on firing rates and correlation coefficients in the perturbative regime

Estimation of the KL divergences and 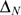 from real data can be hard, in the sense that it takes a large amount of data for them to converge to their true values. In addition, as discussed above, in the section “The prefactors *g_ind_* and *g_pair_*”, there are fluctuations in 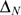 associated with finite subsampling of the full population of neurons. Those fluctuations tend to keep 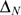 from being purely linear, as can seen, for example, in the blue points in Fig. 5F and 5I. We therefore provide a second set of relationships that can be used to determine whether or not a particular data set is in the perturbative regime. These relationships are between the parameters of the maximum entropy model, the 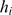 and 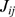, and the mean activity and normalized second order correlation coefficient (the latter defined in Eq. (19) below).

Since the quantity 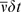 plays a central role in our analysis, we replace it with a single parameter, which we denote 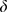,
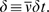
(17)In terms of this parameter, we find (using the same perturbative approach that led us to Eqs. (8–10); see the section “External fields, pairwise couplings and moments,” Methods), that
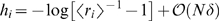
(18a)

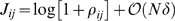
(18b)where 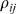, the normalized second order correlation coefficient, is defined in Eq. (16) with 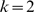; it is given explicitly by
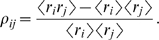
(19)(We don't need a superscript on 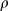 or a subscript on the angle brackets because the first and second moments are the same under the true and pairwise distributions.) Equation (18a) can be reconstructed from the low firing rate limit of analysis carried out by Sessak and Monasson [17], as can the first three terms in the expansion of the log in Eq. (18b).

**Figure 6:**
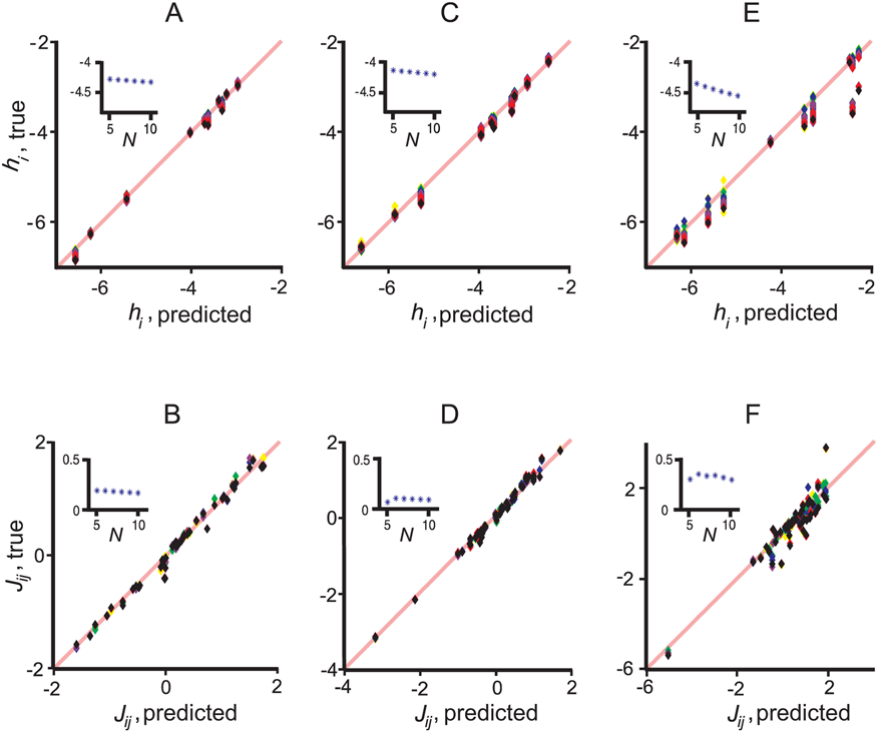
The true external fields and pairwise interactions compared with the predictions of the perturbation expansion. The top row shows the true external fields, 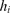, versus those predicted from Eq. (18a), and the bottom row shows the true pairwise interaction, 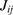, versus those predicted from Eq. (18b). Values of 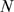 ranging from 5 to 10 are shown, with different colors corresponding to different 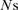. For each value of 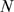, data is shown for 45 realization of the true distribution. Insets show the 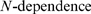 of the mean external fields (top) and mean pairwise interactions (bottom). The three columns correspond exactly to the columns in Fig. 5. (A, B) (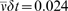). There is a very good match between the true and predicted values of both external fields and pairwise interactions. (C, D) (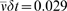). Even though 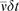 has increased, the match is still good. (E, F) (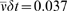). The true and predicted external fields and pairwise interactions do not match as well as the cases shown in (A, B, C, D). There is also now a stronger 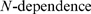 in the mean external fields compared to (A) and (B). The 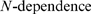 of the pairwise interactions in (F) is weaker than that of the external fields, but still notably stronger than the ones in (B) and (D). This indicates that the perturbative expansion is starting to break down.

**Figure 7:**
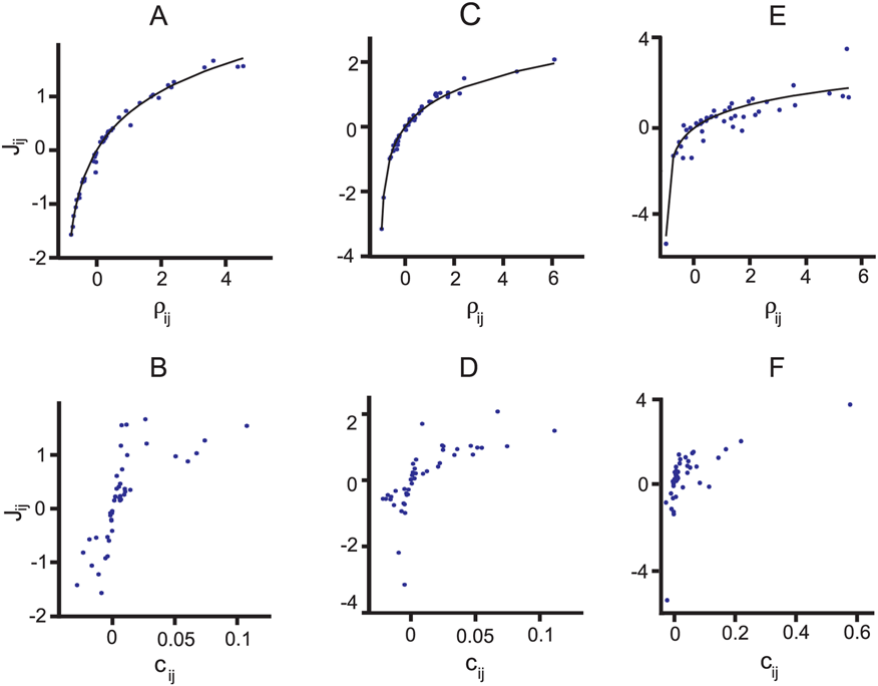
The relation between pairwise couplings and pairwise correlations. This figure shows that there is a simple relation between 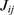 and 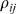, but not between 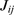 and 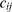. (A, C, E) 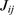 versus the normalized coefficients, 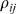 (blue points), along with the predicted relationship, via Eq. (18b) (black line). (B, D, F) 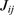 versus the Pearson correlation coefficients, 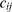, Eq. (26) (blue points). The three columns correspond exactly to the columns in Fig. 5 from left to right; that is, 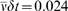 for (A, B), 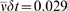 for (C, D), and 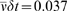 for (E, F). The prediction in the top row (black line) matches the data well, even in the rightmost column.

Equation (18) tells us that the 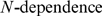 of the 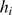 and 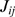, the external fields and pairwise couplings, is very weak. In Fig. 6 we confirm this through numerical simulations. Equation (18b) also provides additional information—it gives us a functional relationship between the pairwise couplings and the normalized pairwise correlations function, 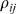. In Fig. 7A–C we plot the pairwise couplings, 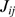, versus the normalized pairwise correlation coefficient, 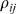 (blue dots), along with the prediction from Eq. (18b) (black line). Consistent with our predictions, the data in Fig. 7A–C essentially follows a line—the line given by Eq. (18b).

A relationship between the pairwise couplings and the correlations coefficients has been sought previously, but for the more standard Pearson correlation coefficient [7],[9],[11]. Our analysis explains why it was not found. The Pearson correlation coefficient, denoted 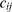, is given by
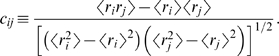
(20)


In the small 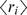 limit—the limit of interest—the right hand side of Eq. (20) is approximately equal to 
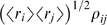
. Because 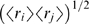 depends on the external fields, 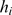 and 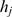 (see Eq. (18a)) *and* there is a one-to-one relationship between 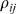 and 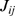 (Eq. (18b)), there can't be a one-to-one relationship between 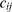 and 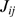. We verify the lack of a relationship in Fig. 7D and 7E, where we again plot 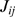, but this time versus the standard correlation coefficient, 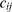. As predicted, the data in Fig. 7D and 7E is scattered over two dimensions. This suggests that 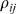, not 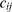, is the natural measure of the correlation between two neurons when they have a binary representation, something that has also been suggested by Amari based on information-geometric arguments [18].

Note that the lack of a simple relationship between the pairwise couplings and the standard correlation coefficient has been a major motivation in building maximum entropy models [7],[11]. This is for good reason: if there is a simple relationship, knowing the 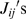 adds essentially nothing. Thus, plotting 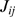 versus 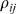 (but not 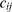) is an important test of one's data, and if the two quantities fall on the curve predicted by Eq. (18b), the maximum entropy model is adding very little information, if any.

As an aside, we should point out that the 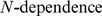 is a function of the variables used to represent the firing patterns. Here we use 0 for no spike and 1 for one or more spikes, but another, possibly more common, representation, derived from the Ising model and used in a number of studies [7],[9],[11], is to use −1 and +1 rather than 0 and 1. This amounts to making the change of variables 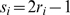. In terms of 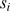, the maximum entropy model has the form 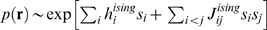 where 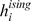 and 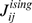 are given by
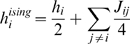
(21a)

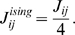
(21b)


The second term on the right side of Eq. (21a) is proportional to 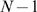, which means the external fields in the Ising representation acquire a linear 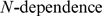 that was not present in our 0/1 representation. The two studies that reported the 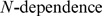 of the external fields [7],[9] used this representation, and, as predicted by our analysis, the external fields in those studies had a component that was linear in 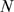.

### Is there anything wrong with using small time bins?

An outcome of our perturbative approach is that our normalized distance measure, 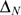, is linear in bin size (see Eq. (10b)). This suggests that one could make the pairwise model look better and better simply by making the bin size smaller and smaller. Is there anything wrong with this? The answer is yes, for reasons discussed above (see the the section “The extrapolation problem”); here we emphasize and expand on this issue, as it is an important one for making sense of experimental results.

The problem arises because what we have been calling the “true” distribution is not really the true distribution of spike trains. It is the distribution assuming independent time bins, an assumption that becomes worse and worse as we make the bins smaller and smaller. (We use this potentially confusing nomenclature primarily because all studies of neuronal data carried out so far have assumed temporal independence, and compared the pairwise distribution to the temporally independent—but still correlated across neurons—distribution [7]–[9],[11]. In addition, the correct name “true under the assumption of temporal independence,” is unwieldy.) Here we quantify how much worse. In particular, we show that if one uses time bins that are small compared to the characteristic correlation time in the spike trains, the pairwise model will not provide a good description of the data. Essentially, we show that, when the time bins are too small, the error one makes in ignoring temporal correlations is larger than the error one makes in ignoring correlations across neurons.

As usual, we divide time into bins of size 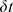. However, because we are dropping the independence assumption, we use 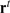, rather than 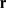, to denote the response in bin 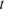. The full probability distribution over all time bins is denoted 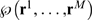. Here 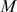 is the number of bins; it is equal to 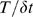 for spike trains of length 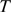. If time bins are approximately independent and the distribution of 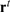 is the same for all 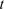 (an assumption we make for convenience only, but do not need; see the section “Extending the normalized distance measure to the time domain,” Methods), we can write
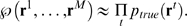
(22)


Furthermore, if the pairwise model is a good one, we have
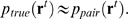
(23)


Combining Eqs. (22) and Eq. (23) then gives us an especially simple expression for the full probability distribution: 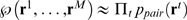.

The problem with small time bins lies in Eq. (22): the right hand side is a good approximation to the true distribution when the time bins are large compared to the spike train correlation time, but it is a bad approximation when the time bins are small (because adjacent time bins become highly correlated). To quantify how bad, we compare the error one makes assuming independence across time to the error one makes assuming independence across neurons. The ratio of those two errors, denoted 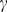, is given by
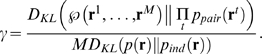
(24)


It is relatively easy to compute 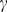 in the limit of small time bins (see the section “Extending the normalized distance measure to the time domain,” Methods), and we find that
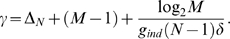
(25)


As expected, this reduces to our old result, 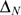, when there is only one time bin (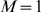). When 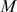 is larger than 1, however, the second term is always at least one, and for small bin size, the third term is much larger than one. Consequently, if we use bins that are small compared to the temporal correlation time of the spike trains, the pairwise model will do a very bad job describing the full, temporally correlated spike trains.

## Discussion

Probability distributions over the configurations of biological systems are extremely important quantities. However, because of the large number of interacting elements comprising such systems, these distributions can almost never be determined directly from experimental data. Using parametric models to approximate the true distribution is the only existing alternative. While such models are promising, they are typically applied only to small subsystems, not the full system. This raises the question: are they good models of the full system?

We answered this question for a class of parametric models known as pairwise models. We focused on a particular application, neuronal spike trains, and our main result is as follows: if one were to record spikes from multiple neurons, use sufficiently small time bins and a sufficiently small number of cells, and assume temporal independence, then a pairwise model will almost always succeed in matching the true (but temporally independent) distribution—whether or not it would match the true (but still temporally independent) distribution for large time bins or a large number of cells. In other words, pairwise models in the “sufficiently small” regime, what we refer to as the perturbative regime, have almost no predictive value for what will happen with large populations. This makes extrapolation from small to large systems dangerous.

This observation is important because pairwise models, and in particular pairwise maximum entropy models, have recently attracted a great deal of attention: they have been applied to salamander and guinea pig retinas [7], primate retina [8], primate cortex [9], cultured cortical networks [7], and cat visual cortex [11]. These studies have mainly operated close to the perturbative regime. For example, Schneidman et al. [7] had 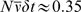 (for the data set described in their Fig. 5), Tang et al. [9] had 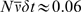 to 0.4 (depending on the preparation), and Yu et al. [11] had 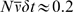. For these studies, then, it would be hard to justify extrapolating to large populations.

The study by Shlens et al. [8], on the other hand, might be more amenable to extrapolation. This is because spatially localized visual patterns were used to stimulate retinal ganglion cells, for which a nearest neighbor maximum entropy models provided a good fit to their data. (Nearest neighbor means 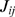 is zero unless neuron 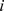 and neuron 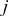 are adjacent.) Our analysis still applies, but, since all but the nearest neighbor correlations are zero, many of the terms that make up 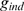 and 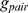 vanish (see Eqs. (42) and (44)). Consequently, the small parameter in the perturbative expansion becomes 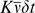 (rather than 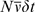), where 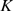 is the number of nearest neighbors. Since 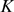 is fixed, independent of the population size, the small parameter will not change as the population size increases. Thus, Shlens et al.may have tapped into the large population behavior even though they considered only a few cells at a time in their analysis. Indeed, they have recently extended their analysis to more than 100 neurons, and they still find that nearest neighbor maximum entropy models provide very good fits to the data [19].

### Time bins and population size

That the pairwise model is always good if 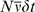 is sufficiently small has strong implications: if we want to build a good model for a particular 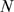, we can simply choose a bin size that is small compared to 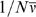. However, one of the assumptions in all pairwise models used on neural data is that bins at different times are independent. This produces a tension between small time bins and temporal independence: small time bins essentially ensure that a pairwise model will provide a close approximation to a model with independent bins, but they make adjacent bins highly correlated. Large time bins come with no such assurance, but they make adjacent bins independent. Unfortunately, this tension is often unresolvable in large populations, in the sense that pairwise models are assured to work only up to populations of size 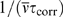 where *τ*_corr_ is the typical correlation time. Given that 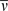 is at least several Hz, for experimental paradigms in which the correlation time is more than a few hundred ms, 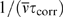 is about one, and this assurance does not apply to even moderately sized populations of neurons.

These observations are especially relevant for studies that use small time bins to model spike trains driven by natural stimuli. This is because the long correlation times inherent in natural stimuli are passed on to the spike trains, so the assumption of independence across time (which is required for the independence assumption to be valid) breaks badly. Knowing that these models are successful in describing spike trains under the independence assumption, then, does not tell us whether they will be successful in describing full, temporally correlated, spike trains. Note that for studies that use stimuli with short correlation times (e.g., non-natural stimuli such as white noise), the temporal correlations in the spike trains are likely to be short, and using small time bins may be perfectly valid.

The only study that has investigated the issue of temporal correlations in maximum entropy models does indeed support the above picture [9]: for the parameters used in that study (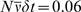 to 0.4), the pairwise maximum entropy model provided a good fit to the data (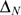 was typically smaller than 0.1), but it did not do a good job modeling the temporal structure of the spike trains.

### Other systems—Protein folding

As mentioned in the Introduction, in addition to the studies on neuronal data, studies on protein folding have also emphasized the role of pairwise interactions [2],[3]. Briefly, proteins consist of strings of amino acids, and a major question in structural biology is: what is the probability distribution of amino acid strings in naturally folding proteins? One way to answer this is to approximate the full probability distribution of naturally folding proteins from knowledge of single-site and pairwise distributions. One can show that there is a perturbative regime for proteins as well. This can be readily seen using the celebrated HP protein model [20], where a protein is composed of only two types of amino acids. If, at each site, one amino acid type is preferred and occurs with high probability, say 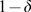 with 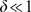, then a protein of length shorter than 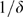 will be in the perturbative regime, and, therefore, a good match between the true distribution and the pairwise distribution for such a protein is virtually guaranteed.

Fortunately, the properties of real proteins generally prevent this from happening: at the majority of sites in a protein, the distribution of amino acids is *not* sharply peaked around one amino acid. Even for those sites that are sharply peaked (the evolutionarily-conserved sites), the probability of the most likely amino acid, 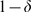, rarely exceeds 90% [21],[22]. This puts proteins consisting of only a few amino acids out of the perturbative regime, and puts longer proteins—the ones usually studied using pairwise models—well out of it.

This difference is fundamental: because many of the studies that have been carried out on neural data were in the perturbative regime, the conclusions of those studies—specifically, the conclusion that pairwise models provide accurate descriptions of large populations of neurons—is not yet supported. This is not the case for the protein studies, because they are not in the perturbative regime. Thus, the evidence that pairwise models provide accurate descriptions of protein folding remain strong and exceedingly promising.

### Open questions

In our analysis, we sidestepped two issues of practical importance: finite sampling and alternative measures for assessing the quality of the pairwise model. These issues are beyond the scope of this paper, but in our view, they are natural next steps in the analysis of pairwise models. Below we briefly expand on them.

Finite sampling refers to the fact that in any real experiment, one has access to only a finite amount of data, and so does not know the true probability distribution of the spike trains. In our analysis, however, we assumed that one did have full knowledge of the true probability distribution. Since a good estimate of the probability distribution is crucial for assessing whether the pairwise model can be extrapolated to large populations, it is important to study how such estimates are affected by finite data. Future work is needed to address this issue, and to find ways to overcome data limitation—for example, by finding efficient methods for removing the finite data bias that affects information theoretic quantities such as the Kullback-Leibler divergence.

There are always many possible ways to assess the quality of a model. Our choice of 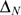 was motivated by two considerations: it is based on the Kullback-Leibler divergence, which is a standard measure of “distance” between probability distributions, and it is a widely used measure in the field [7]–[10]. It suffers, however, from a number of shortcomings. In particular, 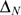 can be small even when the pairwise model assigns very different probabilities to many of the configurations of the system. It would, therefore, be important to study the quality of pairwise models using other measures.

## Methods

### The behavior of the true entropy in the large 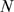 limit

To understand how the true entropy behaves in the large 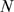 limit, it is useful to express the difference of the entropies as a mutual information. Using 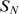 to denote the true entropy of 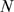 neurons and 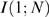 to denote the mutual information between one neuron and the other 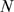 neurons in a population of size 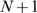, we have
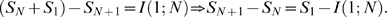
(26)


If knowing the activity of 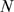 neurons does not fully constrain the firing of neuron 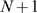, then the single neuron entropy, 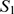, will exceed the mutual information, 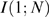, and the entropy will be an increasing function of 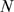. For the entropy to be linear in 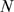, all we need is that the mutual information saturates with 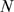. Because of synaptic noise, this is a reasonable assumption for networks of neurons: even if we observed all the spikes from all the neurons, there would still be residual noise associated with synaptic failures, jitter in release time, variability in the amount of neurotransmitter released, stochastic channel dynamics, etc. Consequently, in the large 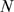 limit, we may replace 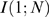 by its average, denoted 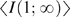. Also replacing 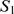 by its average, denoted 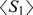, we see that for large 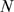, the difference between 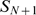 and 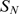 approaches a constant. Specifically,
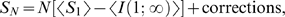
(27)where this expression is valid in the large 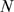 limit and the corrections are sublinear in 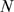.

### Perturbative expansion

Our main quantitative result, given in Eqs. (8–10), is that the KL divergence between the true distribution and both the independent and pairwise distributions can be computed perturbatively as an expansion in powers of 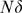 in the limit 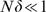. Here we carry out this expansion, and derive explicit expressions for the quantities 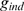 and 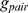.

To simplify our notation, here we use 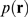 for the true distribution. The critical step in computing the KL divergences perturbatively is to use the Sarmanov-Lancaster expansion [23]–[28] for 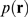,
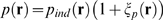
(28)where
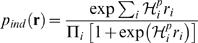
(29a)

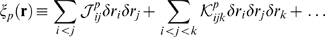
(29b)

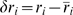
(29c)

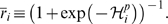
(29d)


This expansion has a number of important, but not immediately obvious, properties. First, as can be shown by a direct calculation,
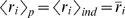
(30)where the subscripts 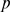 and 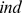 indicate an average with respect to 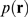 and 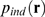, respectively. This has an immediate corollary,
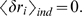



This last relationship is important, because it tells us that if a product of 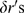 contains any terms linear in one of the 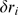, the whole product averages to zero under the independent distribution. This can be used to show that
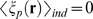
(31)from which it follows that
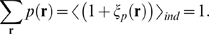



Thus, 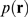 is properly normalized. Finally, a slightly more involved calculations provides us with a relationship between the parameters of the model and the moments: for 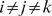,
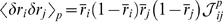
(32a)

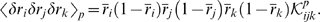
(32b)


Virtually identical expressions hold for higher order moments. It is this last set of relationships that make the Sarmanov-Lancaster expansion so useful.

Note that Eqs. (32a) and (32b), along with the expression for the normalized correlation coefficients given in Eq. (16), imply that
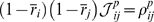
(33a)

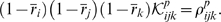
(33b)


These identities will be extremely useful for simplifying expressions later on.

Because the moments are so closely related to the parameters of the distribution, moment matching is especially convenient: to construct a distribution whose moments match those of 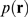 up to some order, one simply needs to ensure that the parameters of that distribution, 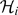, 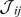, 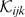, etc., are identical to those of the true distributions up to the order of interest. In particular, let us write down a new distribution, 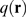,
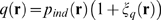
(34a)

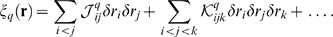
(34b)


We can recover the independent distribution by letting 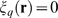, and we can recover the pairwise distribution by letting 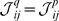. This allows us to compute 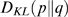 in the general case, and then either set 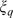 to zero or set 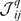 to 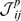.

Expressions analogous to those in Eqs. (31–33) exist for averages with respect to 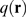; the only difference is that 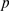 is replaced by 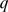.

### The KL divergence in the Sarmanov-Lancaster representation

Using Eqs. (28) and (34a) and a small amount of algebra, the KL divergence between 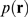 and 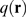 may be written
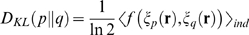
(35)where
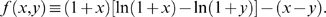
(36)


To derive Eq. (35), we used the fact that 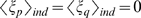 (see Eq. (31)). The extra term 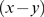 was included to ensure that 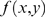 and its first derivatives vanish at 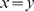, something that greatly simplifies our analysis later on.

Our approach is to Taylor expand the right hand side of Eq. (35) around 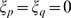, compute each term, and then sum the *whole* series (we do not assume that either 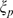 or 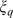 is small). Using 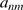 to denote the coefficients of the Taylor series, we have
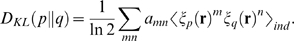
(37)


Note that because 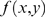 and its first derivatives vanish at 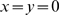, all terms in this sum have 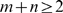.

Because both 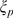 and 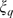 are themselves sums, an exact calculation of the terms in Eq. (37) would be difficult. However, as we show below, in the section “Averages of powers of *ξ_p_* and *ξ_q_*” (see in particular Eqs. (52) and (54)), they can be computed as perturbation expansions in powers of 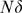, and the result is
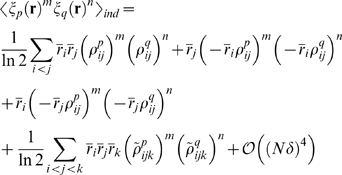
(38)where 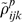 and 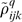 are given by
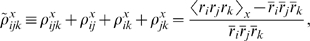
(39)

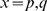
. The last equality in Eq. (39) follows from a small amount of algebra and the definition of the correlation coefficients given in Eq. (16). Equation (38) is valid only when 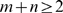, which is the case of interest to us (since the Taylor expansion of 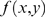 has only terms with 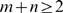).

The important point about Eq. (38) is that the 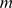 and 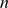 dependence follows that of the original Taylor expansion. Thus, when we insert this equation back into Eq. (37), we recover our original function—all we have to do is interchange the sums. For example, consider inserting the first term in Eq. (38) into Eq. (37),
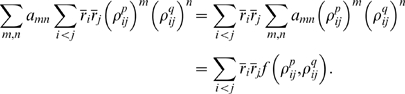



Performing the same set of manipulations on all of Eq. (38) leads to
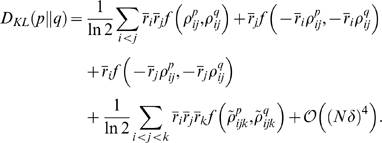
(40)


This expression is true in general (except for some technical considerations; see the section “Averages of powers of *ξ_p_* and *ξ_q_*”); to restrict it to the KL divergences of interest we set 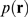 to 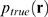 and 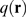 to either 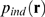 or 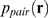. In the first case (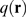 set to 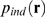), 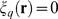, which implies that 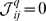, and thus 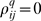. Because 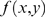 has a quadratic minimum at 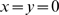, when 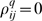, the second two terms on the right hand side of Eq. (40) are 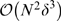. We thus have, to lowest nonvanishing order in 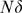,
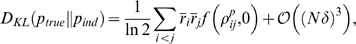
(41)with the 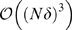 correction coming from the last sum in Eq. (40). Defining
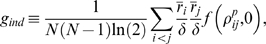
(42)where, recall 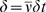, and inserting Eq. (42) into Eq. (41), we recover Eq. (8a).

In the second case (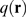 set to 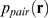), the first and second moments of 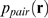 and 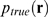 are equal. This implies, using Eq. (32), that 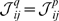, and thus 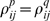. Because 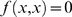 (see Eq. (36)), the first three terms on the right hand side of Eq. (40)—those involving 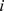 and 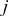 but not 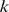
—vanish. The next order term does not vanish, and yields
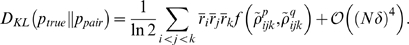
(43)


Defining
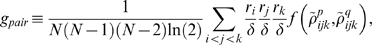
(44)and inserting this expression into Eq. (43), we recover Eq. (8b).

### External fields, pairwise couplings and moments

In this section we derive, to leading order in 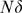, expressions relating the external fields and pairwise couplings of the maximum entropy model, 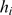 and 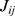, to the first and second moments; these are the expressions reported in Eq. (18). The calculation proceeds along the same lines as in the previous section. There is, though, one extra step associated with the fact that the quadratic term in the maximum entropy distribution given in Eq. (14) is proportional to 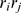, not 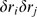. However, to lowest order in 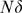, this doesn't matter. That's because
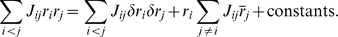
where 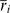 is defined as in Eq. (29d) except with 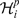 replaced by 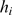, and we used the fact that 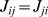. The second term introduces a correction to the external fields, 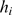. However, the correction is 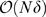, so we drop it. We should keep in mind, though, that our final expression for 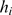 will have corrections of this order.

Using Eq. (14), but with 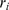 replaced by 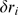 where it appears with 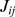, we may write (after a small amount of algebra)
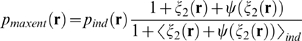
(45)where 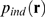 is the same as the function 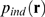 defined in Eq. (29a) except that 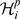 is replaced by 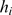, the subscript “*ind*” indicates, as usual, an average with respect to 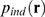, and the two functions 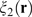 and 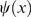 are defined by
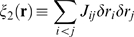
(46)and
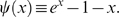
(47)


Given this setup, we can use Eqs. (55) and (56) below to compute the moments under the maximum entropy model. That's because both 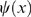 and its first derivative vanish at 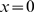, which are the two conditions required for these equations to be valid. Using also the fact that 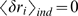, Eqs. (55) and (56) imply that
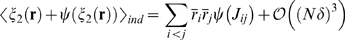
(48a)

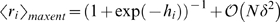
(48b)

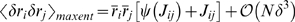
(48c)where the first term in Eq. (48b) came from Eq. (29d) with 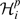 replaced by 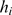, the term “
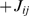
” in Eq. (48c) came from 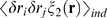, and for the second two expressions we used the fact that, to lowest order in 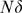, the denominator in Eq. (45) is equal to 1.

Finally, using Eq. (19) for the normalized correlation coefficient, dropping the subscript “*maxent*” (since the first and second moments are the same under the maxent and true distributions), and inverting Eqs. (48b) and (48c) to express the external fields and coupling coefficients in terms of the first and second moments, we arrive at Eq (18).

### Averages of powers of 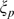 and 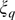


Here we compute 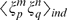, which, as can be seen in Eq. (37), is the key quantity in our perturbation expansion. Our starting point is to (formally) expand the sums that make up 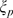 and 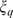 (see Eqs. (29b) and (34b)), which yields
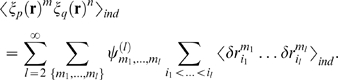
(49)


The sum over 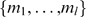 is a sum over all possible configurations of the 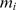. The coefficient 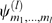 are complicated functions of the 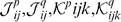, etc. Computing these functions is straightforward, although somewhat tedious, especially when 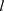 is large; below we compute them only for 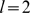 and 3. The reason 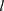 starts at 2 is that 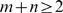; see Eq. (37).

We first show that all terms with superscript 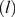 are 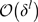. To do this, we note that, because the right hand side of Eq. (49) is an average with respect to the independent distribution, the average of the product is the product of the averages,
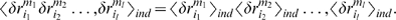
(50)Then, using the fact that 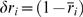 with probability 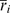 and 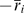 with probability 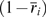 (see Eq. (29c)), we have
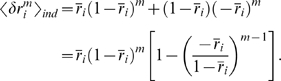
(51)


The significance of this expression is that, for 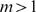, 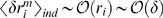, independent of 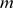. Consequently, if all the 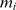 in Eq. (50) are greater than 1, then the right hand side is 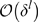. This shows that, as promised above, the superscript 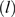 labels the order of the terms.

As we saw in the section “The KL divergence in the Sarmanov-Lancaster representation”, we need to go to third order in 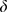, which means we need to compute the terms on the right hand side of Eq. (49) with 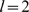 and 3. Let us start with 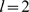, which picks out only those terms with two unique indices. Examining the expressions for 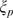 and 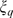 given in Eqs. (29b) and (34b), we see that we must keep only terms involving 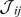, since 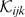 has three indices, and higher order terms have more. Thus, the 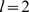 contribution to the average in Eq. (49), which we denote 
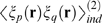
, is given by
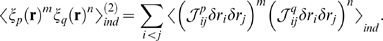



Pulling 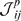 and 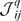 out of the averages, using Eq. (33a) to eliminate 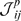 and 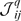 in favor of 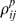 and 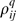, and applying Eq. (51) (while throwing away some of the terms in that equation that are higher than second order in 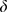), the above expression may be written
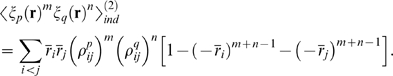
(52)


Note that we were not quite consistent in our ordering with respect to 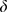, in the sense that we kept some higher order terms and not others. We did this so that we could use 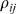 rather than 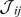, as the former is directly observable.

For 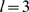 the calculation is more involved, but not substantially so. Including terms with exactly three unique indices in the sum on the right hand side of Eq. (49) gives us
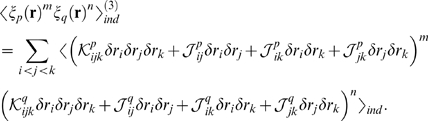
(53)


This expression is not quite correct, since some of the terms contain only two unique indices—these are the terms proportional to 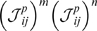
—whereas it should contain only terms with exactly three unique indices. Fortunately, this turns out not to matter, for reasons we discuss at the end of the section.

To perform the averages in Eq. (53), we would need to use multinomial expansions, and then average over the resulting powers of 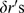. For the latter, we can work to lowest order in 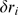, which means we only take the first term in Eq. (51). This amounts to replacing every 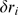 with 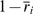 (and similarly for 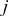 and 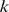), and in addition multiplying the whole expression by an overall factor of 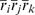. For example, if 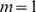 and 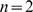, one of the terms in the multinomial expansion is 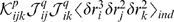. This average would yield, using Eq. (51) and considering only the lowest order term, 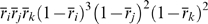.

This procedure also is not quite correct, since terms with only one factor of 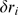, which average to zero, are replaced with 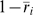. This also turns out not to matter; again, we discuss why at the end of the section.

We can, then, go ahead and use the above “replace blindly” algorithm. Note that the factors of 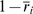, 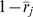 and 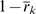 turn 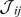 and 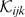 into normalized correlation coefficients (see Eq. (33)), which considerably simplifies our equations. Using also Eq. (39) for 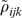, Eq. (53) becomes
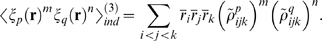
(54)


We can now combine Eqs. (52) and (54), and insert them into Eq. (49). This gives us the first two terms in the perturbative expansion of 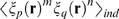; the result is written down in Eq. (38) above.

Why can we ignore the overcounting associated with terms in which an index appears exactly zero or one times? We clearly can't do this in general, because for such terms, replacing 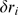 with 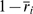 fails—either because the terms didn't exist in the first place (when one of the indices never appeared) or because they averaged to zero (when an index appeared exactly once). In our case, however, such terms do not appear in the Taylor expansion. To see why, note first of all that, because of the form of 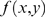, its Taylor expansion can be written 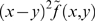 where 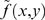 is finite at 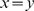 (see Eq. (36)). Consequently, the original Taylor expansion of 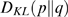, Eq. (37), should contain a factor of 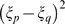; i.e.,
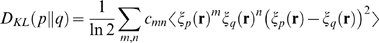
where the 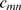 are the coefficients of the Taylor expansion of 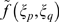. The factor 
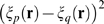
, when expanded, has the form
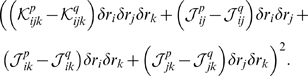



As we saw in the previous section, we are interested in the third order term only to compute 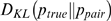, for which 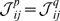. Therefore, the above multiplicative factor reduces to 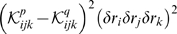. It is that last factor of 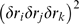 that is important, since it guarantees that for every term in the Taylor expansion, all indices appear at least twice. Therefore, although Eq. (53) is not true in general, it is valid for our analysis.

We end this section by pointing out that there is a very simple procedure for computing averages to second order in 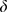. Consider a function 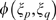 that has a minimum at 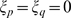 and also obeys 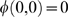. Then, based on the above analysis, we have
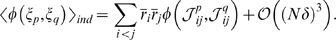
(55)


Two easy corollaries of this are: for 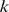 and 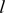 positive integers,
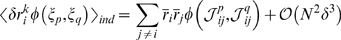
(56a)

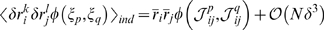
(56b)where the sum in Eq. (56a) runs over 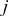 only, and we used the fact that both 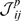 and 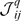 are symmetric with respect to the interchange of 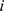 and 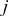.

### Generating synthetic data

As can be seen in Eq. (13), the synthetic data depends on three sets of parameters: 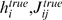, and 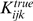. Here we describe how they were generated.

To generate the 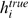, we draw a set of firing rates, 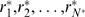, from an exponential distribution with mean 0.02 (recall that 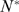, which we set to 15, is the number of neurons in our base distribution). From this we chose the external field according to Eq. (18a),
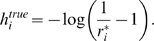



In the perturbative regime, a distribution generated with these values of the external fields has firing rates approximately equal to the 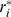; see Eq. (18a) and Fig. 6.

To generate the 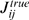 and 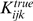, we drew them from Gaussian distributions with means equal to 0.05 and 0.02 and standard deviations of 0.8 and 0.5, respectively. Using non-zero values for 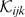 means that the true distribution is not pairwise.

### Bin size and the correlation coefficients

One of our main claims is that 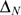 is linear in bin size, 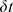. This is true, however, only if 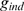 and 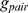 are independent of 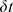, as can be seen from Eq. (10b). In this section we show that independence is satisfied if 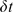 is smaller than the typical correlation time of the responses. For 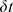 larger than such correlation times, 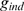 and 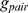 do depend on 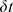, and 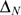 is no longer linear in 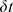. Note, though, that the correlation time is always finite, so there will always be a bin size below which the linear relationship, 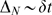, is guaranteed.

Examining Eqs. (42) and (44), we see that 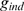 and 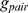 depend on the normalized correlation coefficients, 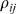 and 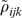 (we drop superscripts, since our discussion will be generic). Thus, to understand how 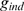 and 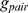 depend on bin size, we need to understand how the normalized correlation coefficients depend on bin size. To do that, we express them in terms of standard cross-correlograms, as the cross-correlograms contain, in a very natural way, information about the temporal timescales in the spike train.

We start with the second order correlation coefficient, since it is simplest. The corresponding cross-correlogram, which we denote 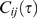, is given by
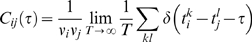
(57)where 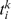 is the time of the *k* ^th^ spike on neuron 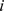 (and similarly for 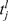), and 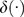 is the Dirac 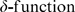. The normalization in Eq. (57) is slightly non-standard—more typical is to divide by something with units of firing rate (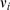, 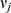 or 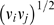), to give units of spikes/s. The normalization we use is convenient, however, because in the limit of large 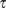, 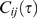 approaches one.

It is slightly tedious, but otherwise straightforward, to show that when 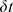 is sufficiently small that only one spike can occur in a time bin, 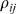 is related to 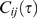 via
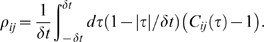
(58)


The (unimportant) factor 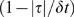 comes from the fact that the spikes occur at random locations within a bin.

Equation (58) has a simple interpretation: 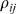 is the average height of the central peak of the cross-correlogram relative to baseline. How strongly 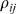 depends on 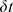 is thus determined by the shape of the cross-correlogram. If it is smooth, then 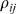 approaches a constant as 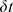 becomes small. If, on the other hand, there is a sharp peak at 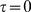, then 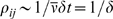 for small 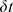, so long as 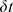 is larger than the width of the peak. (The factor of 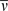 included in the scaling is approximate; it is a placeholder for an effective firing rate that depends on the indices 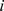 and 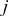. It is, however, sufficiently accurate for our purposes.) A similar relationship exists between the third order correlogram and the correlation coefficient. Thus, 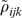 is also independent of 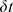 in the small 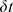 limit, whereas if the central peak is sharp it scales as 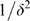.

The upshot of this analysis is that the shape of the cross-correlogram has a profound effect on the correlation coefficients and, therefore, on 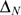. What is the shape in real networks? The answer typically depends on the physical distance between cells. If two neurons are close, they are likely to receive common input and thus exhibit a narrow central peak in their cross-correlogram. Just how narrow depends on the area. Early in the sensory pathways, such as retina [29]–[31] and LGN [32], peaks can be very narrow—on the order of milliseconds. Deeper into cortex, however, peaks tend to broaden, to at least tens of milliseconds [33],[34]. If, on the other hand, the neurons are far apart, they are less likely to receive common input. In this case, the correlations come from external stimuli, so the central peak tends to have a characteristic width given by the temporal correlation time of the stimulus, typically 100 s of milliseconds.

Although clearly both kinds of cross-correlograms exist in any single population of neurons, it is convenient to analyze them separately. We have already considered networks in which the cross-correlograms were broad and perfectly flat, so that the correlation coefficients were strictly independent of bin size. We can also consider the opposite extreme: networks in which the the cross-correlograms (both second and higher order) among nearby neurons exhibit sharp peaks while those among distant neurons are uniformly equal to 1. In this regime, the correlation coefficients depend on 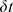: as discussed above, the second order ones scale as 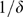 and the third as 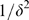. This means that the arguments of 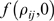 and 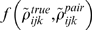 are large (see Eqs. (42) and (44)). From the definition of 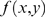 in Eq. (36), in this regime both are approximately linear in their arguments (ignoring log corrections). Consequently, 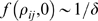 and 
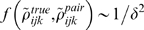
. This implies that 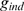 and 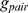 scale as 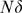 and 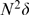, respectively, and so 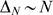, independent of 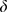. Thus, if the bin size is large compared to the correlation time, 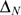 will be approximately independent of bin size.

### Extending the normalized distance measure to the time domain

In this section we derive the expression for 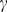 given in Eq. (25). Our starting point is its definition, Eq. (24). It is convenient to define 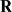 to be a concatenation of the responses in 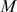 time bins,
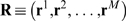
(59)where, as in the section “Is there anything wrong with using small time bins?”, the superscript labels time, so 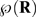 is the full, temporally correlated, distribution.

With this definition, we may write the numerator in Eq. (24) as
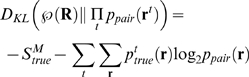
(60)where 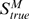 is the entropy of 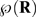, the last sum follows from a marginalization over all but one element of 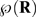, and 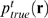 is the true distribution at time 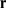 (unlike in the section “Is there anything wrong with using small time bins?”, here we do not assume that the true distribution is the same in all time bins). Note that 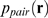 is independent of time, since it is computed from a time average of the true distribution. That time average, which we call 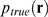, is given in terms of 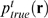 as
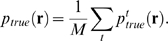



Inserting this definition into Eq. (60) eliminates the sum over 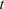, and replaces it with 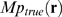. For simplicity we consider the maximum entropy pairwise model. In this case, because 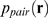 is in the exponential family, and the first and second moments are the same under the true and maximum entropy distributions, we can replace 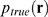 with 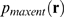. Consequently, Eq. (60) becomes
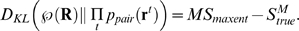



This gives us the numerator in the expression for 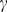 (Eq. (24)); using Eq. (4) to write 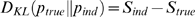, the full expression for 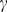 becomes
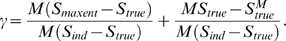
(61)where we added and subtracted 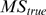 to the numerator.

The first term on the right hand side of Eq. (61) we recognize, from Eq. (6), as 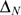. To cast the second into a reasonable form, we define 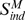 to be the entropy of the distribution that retains the temporal correlations within each neuron but is independent across neurons. Then, adding and subtracting this quantity to the numerator in Eq. (61), and also adding and subtracting 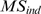, we have
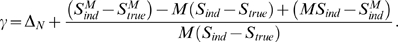
(62)


The key observation is that if 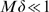, then
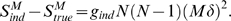



Comparing this with Eqs. (8a) and (9a), we see that 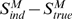 is a factor of 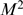 times larger than 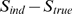. We thus have
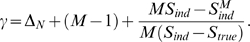
(63)


Again assuming 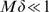, and defining 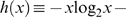 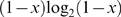, the last term in this expression may be written
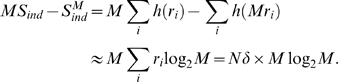
(64)


Inserting this into Eq. (63) and using Eqs. (4), (8a) and (9a) yields Eq. (25).

We have assumed here that 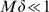; what happens when 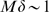, or larger? To answer this, we rewrite Eq. (61) as
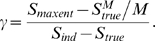
(65)


We argue that in general, as 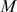 increases, 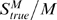 becomes increasingly different from 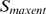, since the former was derived under the assumption that the responses at different time bins were independent. Thus, Eq. (25) should be considered a lower bound on 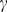.
